# The Role of Tumor/Dendritic Cell Interactions in the Regulation of Anti-Tumor Immunity: The Good, the Bad, and the Ugly

**DOI:** 10.3389/fimmu.2014.00178

**Published:** 2014-04-16

**Authors:** Kristian Michael Hargadon, Timothy N. J. Bullock

**Affiliations:** ^1^Hargadon Laboratory, Department of Biology, Hampden-Sydney College, Hampden-Sydney, VA, USA; ^2^Department of Pathology, University of Virginia, Charlottesville, VA, USA

**Keywords:** tumor, dendritic cell, cancer immunotherapy, tumor immune evasion, cross-presentation, immune regulation

Since the discovery of dendritic cells (DCs) by Ralph Steinman and Zanvil Cohn 40 years ago (1), the role of these cells as critical regulators of immune tolerance versus activation has emerged as one of the most fundamental concepts in the field of immunology. Serving as a link between the innate and adaptive immune systems, DCs exhibit sensitive immune surveillance capabilities that enable their acquisition of antigens from a variety of sources in peripheral tissues, and they possess unique sensory properties and antigen processing machinery that enable their transformation into potent antigen-presenting cells (APCs). Importantly, the outcome (immune tolerance versus activation) of antigen presentation to T cells by DC is dependent on the maturation and activation state of the DC, and significant efforts over the last 20+ years have therefore focused on understanding factors that regulate DC maturation and activation. While their role in self-tolerance and the activation of T cell immunity to foreign pathogens has long been appreciated, more recently DCs have also been shown to play important roles in the regulation of anti-tumor immune responses. Since this time, considerable efforts have been placed on understanding many facets of tumor-associated DC, including: the induction, regulation, and maintenance of anti-tumor immunity by DC; tumor-associated interference with these processes to subvert anti-tumor immunity; and the application of this knowledge to develop therapeutic strategies for improving DC-mediated anti-tumor immune responses. In this collection of articles, we highlight our current understanding of the role played by DC in anti-tumor immunity and focus attention on important questions that remain to be answered in the field as we aim to improve the immunogenicity of tumor-associated DC and the outcome of DC-mediated anti-tumor immune responses in the future.

We begin this research topic with an Opinion article by Rolf Zinkernagel (2) and a responding Commentary from Anne Hosmalin (3), who offer opposing views on cross-presentation of tumor antigen by DC that we believe will generate interesting and thoughtful discussion. These articles are followed by a contribution from Schiavoni et al. (4) reviewing the major subsets of DC that have been implicated in cross-presentation and the role of type I IFN in enhancing DC-mediated cross-priming of anti-tumor CD8+ T cell responses. Research topic co-editor Kristian Hargadon then reviews the various levels at which tumor cells, tumor-derived factors, and tumor-associated cells in the milieu of the tumor microenvironment can interfere with DC function (5). Mechanistic insights into tumor-altered differentiation of DC precursors, tumor-associated suppression of DC maturation and activation, and tumor-induced development of regulatory DC with immunosuppressive function are highlighted, as are recent immunotherapeutic strategies that have been designed to prevent or overcome tumor-associated DC dysfunction and enhance the quality of anti-tumor immune responses. Co-editor Timothy Bullock further examines the metabolic changes that occur in DC during their maturation and discusses how dysregulated metabolism, particularly at the level of glycolysis and fatty acid metabolism, in tumor-associated DC may also impede maturation and contribute to the diminished immune stimulatory function of these cells (6). The impact of tumors on DC maturation is also explored by Dudek et al. (7), who describe the complexity of DC maturation status in the context of tumors, where the typical dichotomy of immature versus mature DC that regulate immune tolerance versus activation against clearly “self” or “non-self” antigen is less obvious. The authors describe a continuum of DC maturation states reported in the context of tumors that include not only the classical immature, tolerogenic DC and mature, immunogenic DC but also semi-mature DC which express low or even moderate levels of costimulatory molecules but which produce minimal stimulatory cytokines and therefore potentiate either tolerogenic or pro-tumorigenic responses. Studies that have identified factors (cytokines/chemokines, cell death modalities, and cancer cell-derived danger signals) regulating tumor-associated DC function are highlighted, as is the ability of anti-cancer therapeutic agents to influence and modulate the maturation states of DC. Additional discussion of this topic is provided by Ott and Bhardwaj (8), who speculate how tumor cell death resulting from MAPK pathway inhibition might enhance cross-presentation by DC in BRAF^V600^ mutant melanoma patients, and by Palombo et al. (9), who describe various danger-associated molecular patterns (DAMPs) released during immunogenic cancer cell death that stimulate inflammatory DC to activate tumor-specific CD8+ T cell responses. This latter Perspective article also discusses evidence for chemotherapy-associated induction of immune responses, particularly against antigens derived from proteins involved in stress pathways that are normally sequestered in healthy cells and therefore are not typically processed or presented to T cells. The authors suggest that such tumor-specific T cells are likely to be useful tools for identifying novel immunogenic tumor-specific antigens as these T cells can be isolated and “interrogated” with purified tumor proteins to assess which antigens are associated with high responsiveness. As researchers consider how cancer cell death influences immune responses in patients, and with renewed interest in the potential of combining traditional cancer therapy and immunotherapy (once thought to be mutually exclusive approaches to cancer treatment), these articles highlight the need to better understand how DC respond (particularly at the level of cytokine secretion) following cancer therapies that induce tumor cell death. Such knowledge will elucidate whether these approaches induce immunogenic versus tolerogenic cell death and promote development of semi-mature versus mature DC, and these insights will have significant implications for optimizing strategies to promote robust anti-tumor immune activation.

A recurring theme in many of the articles presented herein is that tumor immune evasion arises not only from a simple failure of the tumor microenvironment to support DC maturation but also from an active recruitment and exploitation by tumors of immature, tolerogenic DC that suppress adaptive responses. Seliger and Massa (10) review mechanisms by which tumor-derived soluble and membrane-bound factors alter myeloid and plasmacytoid DC function, including effects of these molecules on antigen processing and presentation by DC, T cell stimulatory capacity of DC, migration of DC to tumor-draining lymph nodes, and DC survival. Tesone et al. (11) discuss how tumor-altered myelopoiesis shifts differentiation of myeloid precursors from a DC-committed lineage to lineages with immunosuppressive functions such as myeloid-derived suppressor cells (MDSCs) and tumor-associated macrophages, and these authors also focus on suppressive mechanisms that prevent DC maturation or that induce a switch from immunostimulatory to regulatory DC during tumor progression. Emphasis is also placed on specific recruitment of regulatory DC to tumors by tumor- and stroma-derived chemoattractants and how these tumor-infiltrating DC contribute to the overall suppressive nature of the tumor microenvironment. Vasaturo et al. (12) highlight specifically the negative immune regulation exhibited by PD-1, CTLA-4, and other co-inhibitory molecules and their receptors expressed on cells in the tumor microenvironment, including regulatory DC. These authors bring to light how such interactions hamper not only the induction of anti-tumor immunity by tumor-associated, tolerogenic DC that migrate to draining lymph nodes but also the effector activity of T cells that may have been activated appropriately in secondary lymphoid organs but whose effector function is subject to negative regulation following infiltration of tumors expressing co-inhibitory molecules. While the authors discuss the potential of manipulating costimulatory and co-inhibitory molecule expression in tumors and associated cells as a means of shifting the milieu of the tumor microenvironment from an immunosuppressive state to an immunostimulatory one, they also address the potential limitations of non-specifically administering immune checkpoint inhibitors that may result in autoimmune activation, thus underscoring the need to better understand ways of fine-tuning immune regulation by these molecules and of targeting them in a cell-specific fashion.

Because of the potential of DC to serve as both targets of and delivery agents for tumor immunotherapies, significant efforts have been focused on how best to utilize these cells in the treatment of cancer. Gallois and Bhardwaj (13) review mechanisms by which tumors, Tregs, and immunosuppressive myeloid cells impair DC function and discuss how interventions that aim to combat the suppressive tumor microenvironment can improve the clinical benefit of therapies involving *ex vivo*-generated DC or *in vivo*-targeted DC. In addition to highlighting the need to better understand tumor microenvironmental factors that should be targeted to improve the efficacy of DC-associated immune stimulation in cancer patients, the authors identify a variety of other factors that must further be studied to optimize these therapies, including mechanisms of antigen delivery to endogenous DC; the frequency, route, and site of DC vaccination; methods of DC activation both *ex vivo* and *in vivo*; and the particular DC subsets that should be employed or targeted during immunization. These and other factors are considered more specifically in the context of DC-based therapies in acute and chronic myeloid leukemia patients by Schürch et al. (14), in ovarian cancer patients by Goyne and Cannon (15), and in metastatic melanoma patients by van de Ven et al. (16). Additionally, Ott and Bhardwaj (8) offer insights into the impact of MAPK pathway inhibition on DC activation in melanoma patients carrying the BRAF^V600^ mutation. Finally, Toubai et al. (17) review the roles of both host and donor DC in the induction of graft-versus-host disease and graft-versus-tumor effect following allogeneic hematopoietic stem cell transplantation, and the authors discuss strategies for, and challenges to, uncoupling these two processes as a means of maximizing anti-tumor immunity while minimizing autoimmune reactivity. It is clear from the work summarized in these articles that while this field has moved rapidly in recent years, much remains to be learned to optimize DC-related immune therapies for cancer. Moving forward, it is likely that combinatorial approaches that aim both to block immune inhibitory pathways and to promote immune stimulation will ultimately offer the greatest promise for successful DC-based cancer therapies. For instance, while the success of checkpoint blockade therapy has generally been limited to situations where T cells are already infiltrating tumors, therapies that also target DC, either endogenously or via vaccination, will likely promote anti-tumor T cell activation and therefore increase the proportion of patients for which checkpoint blockade is a viable option.

We conclude this collection of articles with a review by Chmielewski et al. (18), who describe MHC- and APC-independent immunotherapy using chimeric antigen receptor (CAR)-redirected T cells as an alternative to DC-based therapies and traditional adoptive T cell transfer therapy. In light of the tumor-associated suppression of DC described herein and the requirement for adequate expression of MHC molecules by both DC and tumor cells to achieve successful anti-tumor immunity, CAR-redirected T cell therapy has the potential to offer unique advantages over traditional immunotherapies by allowing: (1) the targeting of not only peptides but also carbohydrates and inorganic compounds expressed on tumor cells and (2) the inclusion of intracellular costimulatory molecule signaling domains in chimeric receptors that overcomes the limitation inherent in conventional T cell recognition of tumor cells or tumor-altered DC that typically lack or express low levels of these molecules needed for T cell activation. Furthermore, although CAR-redirected T cells bypass the need for stimulation by DC, it is interesting to speculate that in addition to their direct anti-tumor activity, these T cells might also be useful for the licensing of DC in the tumor microenvironment, thereby indirectly leading to more robust endogenous anti-tumor immune responses as well.

As the intricacies of DC biology and the influence of tumors on DC phenotype and function continue to be uncovered, additional insights into the role of these cells in the induction, regulation, and maintenance of anti-tumor immune responses will continue to shed light on mechanisms of tumor immune escape and inform the design of novel therapies to enhance anti-tumor immunity. It is our hope that the advances highlighted herein and the questions raised for future consideration will generate additional discussion, drive experimental inquiry, and bring into focus the significance of tumor/DC interactions and their impact on overall anti-tumor immunity. Such emphasis is sure to bring rapid advancements in this field and will ultimately lead to the development of more effective cancer immunotherapies and improved clinical outcome in cancer patients in the future.

## Conflict of Interest Statement

The authors declare that the research was conducted in the absence of any commercial or financial relationships that could be construed as a potential conflict of interest.
